# Larvicidal activity of phytosteroid compounds from leaf extract of *Solanum nigrum* against *Culex vishnui* group and *Anopheles subpictus*

**DOI:** 10.1186/s13104-017-2460-9

**Published:** 2017-03-23

**Authors:** Anjali Rawani, Anushree Singha Ray, Anupam Ghosh, Mary Sakar, Goutam Chandra

**Affiliations:** 1 Mosquito, Microbiology and Nanotechnology Research Units, Parasitology Laboratory, Department of Zoology, The University of Burdwan, Golapbag, Burdwan, West Bengal 713104 India; 2 Department of Zoology, Bankura Christian College, Bankura, West Bengal 722101 India; 3 Department of Zoology, University of Gour Banga, Malda, West Bengal 732103 India; 4 Department of Biochemistry, Indian Institute of Science, Bangaluru, Karnataka 560012 India

**Keywords:** *Solanum nigrum*, *Culex vishnui* group, *Anopheles subpictus*, Mosquito control, Phytosteroid

## Abstract

**Background:**

Vector control is facing a menace due to the appearance of resistance to synthetic insecticides. Insecticides of plant origin may provide appropriate substitute biocontrol techniques in the future. The present study was carried out to investigate the bio control potentiality of active ingredient isolated from chloroform: methanol (1:1 v/v) extract of mature leaves of *Solanum nigrum* L. (Solanaceae) against early 3rd instar larvae of *Culex vishnui* group (comprising of *Cx. vishnui* Theobald, *Cx. pseudovishnui* Colless and *Cx. tritaeniorhynchus* Giles) and *Anopheles subpictus* Grassi. *S. nigrum* is a common plant distributed in many parts of India with medicinal properties.

**Methods:**

Bioactive compound isolated from chloroform: methanol (1:1 v/v) extract of mature leaves of *S. nigrum* was (25, 45, 60 mg/L) tested against early 3rd instar larvae of *Cx. vishnui* group and *An. subpictus*. The lethal concentration was determined by log probit analysis. The chemical nature of the active substance was also evaluated following gas chromatography–mass spectroscopy (GC–MS) and infrared (IR) analysis. The compound was also studied on non target organisms such as *Daphnia* sp. and *Diplonychus annulatum*.

**Results:**

TLC spot having R_f_ value of 0.94 (R_f_ = 14.1/15 = 0.94) showed larvicidal activity. In a 72 h bioassay experiment, mortality rate at 60 mg/L was significantly higher (P < 0.05) than those at 25 and 45 mg/L against early 3rd instar. Result of log-probit analysis (at 95% confidence level) revealed that LC_50_ and LC_90_ values gradually decreased with the exposure period showing the lowest value at 72 h of exposure. A clear dose-dependent mortality was observed, as the rate of mortality (Y) was positively correlated with the concentration (X) having regression coefficient value close to one in each case. The compound was found to be eco-friendly as it did not show any adverse effect to the studied non target organisms. Chemical characterization (GC–MS and IR analyses) of the active ingredient revealed the presence of phytosteroid compounds responsible for mosquito larvicidal activity.

**Conclusion:**

Leaf extract of *S. nigrum* has great potential as bio control agent against *Cx. vishnui* group and *An. subpictus*. In near future the isolated bioactive phytochemical could be used as a source of an effective mosquitocidal agent.

## Background

Mosquitoes are the most important single group of insects in terms of public health importance, which are responsible for spreading a number of serious diseases, such as malaria, filariasis, dengue, Japanese encephalitis, etc. and generate a huge economic loss; both in terms of healthcare costs and less productivity [1]. *Anopheles subpictus* Grassi transmits malaria in the rural areas of India [2–4]. Malaria afflicts 36% of the world’s population, i.e., 2020 million in 107 countries and territories situated in the tropical and subtropical regions. In the South East Asian Region, 1.2 billion (85.7%) are exposed to the risk of malaria, most of whom live in India [5]. Japanese encephalitis (JE) is a fatal and common disease in India [6]. The disease is caused by JE virus (JEV), which belongs to the family Flaviviridae and transmitted by *Culex vishnui* group (comprising of *Cx. vishnui* Theobald, *Cx. pseudovishnui* Colless and *Cx. tritaeniorhynchus* Giles) which are the main vectors of JE in different parts of India.

Mosquito control is a vital public health practice throughout the world, especially in the tropics. The most practicable way for reducing incidences of mosquito borne diseases is by controlling mosquito immatures in their breeding sites. Synthetic organic insecticides have been used for several decades in controlling pests and vectors of various human diseases as they have a quick knock down effect. But their indiscriminate use resulted in several problems such as environmental hazards, elimination of natural enemies, toxic residues in food, and also produced insecticidal resistance in major vector species [7]. To overcome from these problems there is an insistent need for search and development of new insecticides which do not have any ill effects on non-target population, easily available as well as cost effective and are easily degradable [8]. Phytochemicals are stored by plants mainly as secondary metabolites that serve as a mean for the defense mechanisms of the plants. Secondary metabolites such as alkaloids, steroids, terpenoids, tannins and flavonoids from different plants have been reported earlier for their insecticidal properties [9]. The plant products or plant-derived compounds are promising alternatives to synthetic insecticides in controlling insect pests of medical importance as these are environmentally safe, biodegradable, of low cost and may be produced using indigenous methods, for vector control [10–14] and can be used with minimum care by individual and communities [15]. Some herbal products such as nicotine obtained from tobacco leaves; anabasine and lupinine, the alkaloids extracted from Russian weed, *Anabasis aphylla* [15], rotenone from *Derris eliptica* [16] and pyrethrums from *Chrysanthemum cinererifolium* flowers [17] have been used as natural insecticides earlier. Kishore et al. [18]. and Ghosh et al. [19]. have also reviewed the present status on the efficacy of plant extracts in the mosquito control study.

*Solanum nigrum* L. commonly known as Makoi or black nightshade usually grows as herb in moist habitats in different kinds of soils. It is 25–100 cm tall, erect annual herb, pubescent with simple hairs. Stems are often angular, sparsely-pubescent. The fruits are dull black, globose, 8–10 mm in diameter. The leaves are ovate, the bases are cuneate, 4–10 and 3–7 cm wide, pubescent, coarsely dentate, the apex is obtuse [20]. The medicinal property of this plant includes anti-inflammatory, antioxidant, antipyretic, antitumor, antiulcerogenic, antinociceptive, cancer chemopreventive, immunomodulatory and hepatoprotective effects [21, 22]. Previously it was reported that an ethyl acetate solvent extract of mature leaves of this plant provided very efficient mosquito larval control [23]. Rawani et al. [23] reported the activity of different solvent extract against early 3rd instars larvae of *Cx. quinquefasciatus*. Highest activity was observed in the ethyl acetate extract, from which glucosinolated compound have been isolated [24]. Chloroform: methanol (1:1 v/v) extract showed second highest mortality after ethyl acetate extract. The present work is an extension of that result to find out active ingredient from chloroform: methanol (1:1 v/v) extract.

The objective of the present study was to isolate the bioactive compounds, if any, from chloroform: methanol (1:1 v/v) extract of mature leaves of *S. nigrum* responsible for larvicidal activity against *Culex vishnui* group and *Anopheles subpictus.*

The study further includes the impact of bioactive principle on *Daphnia* and predatory water-bug as non-target organisms present in the natural habitats of mosquito. The chromatographic, spectroscopic and GC–MS analyses of the active fraction of chloroform: methanol (1:1 v/v) extract was also done for Chemical characterization of the active ingredients responsible for larval toxicity.

## Methods

### Test mosquitoes

The present study was conducted at Burdwan (23°16′ N, 87°54′ E), West Bengal, India. Larvae of *Cx. vishnui* group and *An. subpictus* were collected from nearer rice fields. They were kept separate in different plastic trays and fed with a mixture of dog biscuits and yeast powder at the ratio of 3:1. The larvae were kept free from exposure to pathogens, insecticides, or repellants and maintained at 25–30 °C. The transformed pupae were separated manually with a glass dropper into a glass beaker (500 mL) filled with normal tap water. The beaker was kept in glass cages for emergence of adult mosquitoes. A cotton ball soaked in 10% glucose solution was used for glucose meal of adult mosquitoes and was periodically blood fed on immobilized pigeon. Eggs laid were similarly reared and 1st generation laboratory bred larvae were used for bioassay and control experiments.

### Preparation of solvent extract

We harvested fresh mature leaves of *S. nigrum* during study period (June–July 2012) from outskirts of Burdwan (23°16′ N, 87°54′ E) having voucher specimen No. GCZAR-1 (the herbarium was deposited in the Department of Zoology, The University of Burdwan, West Bengal, India) and authenticated by Dr. Ambarish Mukherjee, Professor of Botany, The University of Burdwan, West Bengal, India. The leaves were initially rinsed with distilled water, dried on paper towel and then shade dried for 7–8 days. The dried leaves were cut into small pieces and 200 gm of them were put in the thimble of Soxhlet apparatus. 2000 ml of chloroform: methanol (1:1 v/v) was then loaded into the still pot. The extraction period was 72 h and the temperature was <40 °C. The extract was collected in a beaker and evaporated in a rotary evaporator (RV8. IKA). Then the sample was lyophilized to get powdered samples for the further bioassay experiment.

### Column chromatography analysis

The column was made grease free using 5% potassium hydroxide (KOH) in ethanol followed by washing with absolute alcohol and then dried with a dryer. Eighty grams of silica gel for column (Merck, India) was taken on a dry filter paper. The column was packed with the silica gel. Then the dried 10 gm sample obtained from chloroform: methanol (1:1 v/v) solvent extract was added in the column along with 5 ml of chloroform: methanol (1:1 v/v). Then the column was eluted with single and mixtures of organic solvents with increased polarity like petroleum ether, petroleum ether: benzene (1:1 v/v), benzene, benzene: chloroform (1:1 v/v), chloroform, chloroform: methanol (1:1 v/v), methanol, methanol: acetone (1:1 v/v), acetone, acetone: absolute alcohol and absolute alcohol. The flow rate was 2 ml/min and each of the fractions was collected in separate test tubes. After preparative Thin Layer Chromatography, fractions with similar Rf values were combined in a single test tube.

### Thin-layer chromatography (TLC) analysis

The bioactive fractions were monitored by thin-layer chromatography on silica gel “G” (Merck, India) coated (0.5 mm thickness) plates using chloroform: methanol (1:1 v/v) as a mobile phase. After progress, plates were dried in the air and then placed in an iodine chamber (21 × 21 × 9 cm) for 1 min. The plate was removed and the main band appeared on the fractions of similar Rf values were mixed together and used as apparently purified compound. The compounds having mosquitocidal effect were further subjected to one-dimensional preparative TLC using solvent system chloroform: methanol (1:1 v/v) as mobile phase. The band containing bioactive toxic principle was detected by keeping the plate in the iodine vapor chamber.

The distance of the run of the developing solvent from the bottom of the plate was measured and the run of the sample spot was also measured. The Rf value was then calculated using formula:

$${\text{Rf}} = {\text{distance}}\,{\text{of}}\,{\text{the}}\,{\text{spot}}\,{\text{centre}}\,{\text{from}}\,{\text{the}}\,{\text{start}}\,{\text{point}}/\,{\text{distance}}\,{\text{of}}\,{\text{the}}\,{\text{solvent}}\,{\text{run}}\,{\text{from}}\,{\text{the}}\,{\text{start}}\,{\text{point}}.$$

### Bioassay with active ingredients

The said band was scrapped from TLC plates and dissolved in 20 ml of absolute alcohol and heated in a water bath (60–65 °C) for 5 min. Clear solutions were taken in separate conical flasks discarding the precipitate including silica gel. After evaporation of alcohol, the solid mass present at the bottom of the conical flask was scrapped and weighed. The fraction was dissolved in distilled water to make different concentrations with the help of micropipette. During the experiment, stock solutions were prepared having concentrations of 25, 45, 60 mg/L. From those stock solutions, 100 ml were taken in each experiment. For the bioassay experiment, 25 third instar larvae of *Cx. vishnui* group and *An. subpictus* were introduced separately into different Petri dishes containing graded nominal concentrations of active ingredient (25, 45, 60 mg/L). All the larval instar were taken in respective Petri-dishes and 20 mg of larval food (powdered mixture of dog biscuits and dried yeast powder in the ratio of 3:1.) was added per Petri-dish. The experiment was kept in a 12 h light/dark setting. The mortality rate was recorded after 24, 48 and 72 h of post-exposure [25]. The data on mortality in 48 and 72 h were expressed by the addition of the mortality at 24 and 48 h, respectively. Dead larvae were identified when they failed to move after probing with a needle in the siphon or cervical region. The experiments were conducted on three different days with 3 replicates on each day for each instar larvae (n = 9) at 25–30 °C and 80–90% relative humidity. A set of control experiment (without having the test solution) using tap water for each instar larvae was also run parallel on each day of the experiment (n = 3).

### IR and GC–MS analyses of bio active principle

A portion of dried sample containing active ingredient was subjected to infrared (IR) spectroscopy. For FTIR analysis, the sample was kept in vacuum desiccators over potassium hydroxide (KOH) pellets for 48 h, and then IR spectral analyses were carried out on a Perkin-Elmer FT-IR Spectrometer (Model: Spectrecee RX1) using potassium bromide (KBr) plates. GC–MS analysis was carried out an SHIMADZU QP 2010T which comprised of an auto sampler and gas chromatography interfaced to a mass spectrometer (GC–MS) instrument employing the following condition: capillary column—624 ms (30 m × 0.32 mm × 1.8 m) operating in an electronic mode at 70 eV; helium (99.999%) was used as the carrier gas at a constant flow of 1.491 ml/min and injection volume of 1.0 ml, injector temperature was 140 °C; ion source temperature of 200 °C. The oven temperature was programmed from 45 °C. Mass spectra were taken at 70 eV.

### Effect on non target organisms

Effect of bioactive compound isolated from mature leaves of *S. nigrum* was tested against non-target organisms (NTO) including *Daphnia* sp. and *Diplonychus annulatum* (predatory water-bug). For acclimation to the laboratory, each of them was kept in an environment similar to their natural habitat. As per the procedure used by Suwannee et al. [26], the non targets were exposed to LC_50_ (at 24 h for early 3rd instar larvae) of the bioactive principle. Twenty five *Daphnia* sp. were placed in 200 ml pond water in a 500 ml beaker. Twenty five early 3rd instar nymph of *Diplonychus annulatum* were kept in pond water in a 12.6 × 10 × 6 inch plastic tray. Numbers of dead NTO were recorded after 24, 48 and 72 h of exposures and percentage mortality was recorded. The experiments were conducted on three different days with 3 replicates on each day for each organism (n = 9) was run parallel. A set of control (without having the test solution) was also run parallel on each day of the experiment (n = 3) and average mortality rates were tabulated.

### Statistical analysis

The percentage mortality observed (M%) was corrected using Abbott’s formula [27] during the observation of the larvicidal potentiality of plant extract. Statistical analysis of the experimental data was performed using the computer software Statplus 2007 and MS EXCEL 2003 to find the regression equations (*Y* = mortality; *X* = concentrations) and regression coefficient values. Probit analysis was done by Statplus 2007 software to find out LC_50_ and LC_90_ values. Completely randomized three-way factorial ANOVA using different concentrations, different mosquito species and hours as variables was performed by SPSS 11.0. In bioassay experiment, nominal values are used for statistical analysis as the actual exposed concentrations are about 10% of the nominal values.

## Result

In the laboratory bioassay, TLC spot having R_f_ value of 0.94 (R_f_ = 14.1/15 = 0.94) showed larvicidal activity. Susceptibility of early 3rd instar larvae of *Cx. vishnui* group and *An. subpictus* to different concentrations of bioactive compound isolated from mature leaves of *S. nigrum* are presented in Table 1. Mortality rate at 60 mg/L was significantly higher (P < 0.05) than those at 25 and 45 mg/L against early 3rd instar. Result of log-probit analysis (at 95% confidence level) revealed that LC_50_ and LC_90_ values gradually decreased with the exposure period showing the lowest value at 72 h of exposure. A clear dose-dependent mortality was observed, as the rate of mortality (Y) was positively correlated with the concentration (X) having regression coefficient value close to one in each case (Table 2). The result of the three-way factorial ANOVA (Table 3) of different mosquito species carried out at different concentrations and different time interval revealed significant differences in larval mortality (P < 0.05). Further by Tukey’s and Duncan test, comparison of mean percentage mortality was done with standard error and their upper and lower bound at the 95% confidence level and the values were significant at P < 0.05 (Table 4).

**Table 1:** Mean larval mortality of early 3rd instar larvae of *Culex vishnui* group and *Anopheles subpictus* to different concentrations of bioactive compound isolated from mature leaves of *Solanum nigrum*

Mosquito species	Concentration (mg/L)	Per cent larval mortality (Mean ± SE)		
		24 h	48 h	72 h
*Cx. vishnui* group	25	62.68 ± 0.67	68.00 ± 0.58	84.00 ± 0.58
	45	76.00 ± 1.15	80.00 ± 1.15	85.32 ± 1.45
	60	80.00 ± 1.15	92.00 ± 0.58	97.32 ± 0.33
	Control	0.00 ± 0.00	0.00 ± 0.00	0.00 ± 0.00
*An. subpictus*	25	68.00 ± 0.58	85.32 ± 1.20	90.68 ± 0.88
	45	81.32 ± 1.20	88.00 ± 1.15	97.32 ± 0.33
	60	86.68 ± 1.20	94.68 ± 0.88	100 ± 0.00
	Control	0.00 ± 0.00	0.00 ± 0.00	0.00 ± 0.00

**Table 2:** Probit and regression analyses of mortality rates of early 3rd instar larvae of *Culex vishnui* group and *Anopheles subpictus* to different concentrations of bioactive compound isolated from mature leaves of *Solanum nigrum*

Mosquito type	Time of exposure (h)	LC_50_ (mg/L)	LC_50_ (mg/L) LCL–UCL	LC_90_ (mg/L)	Regression equation	R value
*Cx. Vishnui* group	24	14.48	0.28–23.79	121.09	Y = 0.12x + 12.75	0.77
	48	15.89	5.37–22.48	61.40	Y = 0.17x + 12.62	0.90
	72	5.64	0.00–14.68	44.33	Y = 0.08x + 18.55	0.63
*An. subpictus*	24	13.21	1.15–21.36	76.27	Y = 0.13x + 13.81	0.79
	48	9.93	0.25–53.38	41.81	Y = 0.06x + 19.52	0.51
	72	3.68	0.56–16.46	24.74	Y = 0.26x + 84.30	0.98

*LC* lethal concentration, *LCL* lower confidence limit, *UCL* upper confidence limit, *R* regression coefficient value

**Table 3:** Three way ANOVA of mortality rates of different mosquito species, different hours of exposure and concentration as variables

Source of variation	Sum of squares	*df*	Mean square	F value	P value
Mosquito species (MS)	46.29	1	46.29	18.12	0.001*
Concentration (C)	132.48	2	66.24	25.92	0.002*
Hours (H)	156.48	2	78.24	30.62	0.003*
MS × C	5.15	2	2.57	1.00	0.375 (NS)
MS × H	1.82	2	0.91	0.36	0.703 (NS)
C × H	10.08	4	2.52	0.98	0.427 (NS)
MS × C × H	9.41	4	2.35	0.92	0.462 (NS)
Residual	92	36	2.56		
Total	453.7	53			

* Significant at P < 0.05

*NS* not significant

**Table 4:** Comparison of mean percentage mortality, standard error and their upper and lower bound at 95% confidence level By Tukey’s and Duncan test

Mosquito species	Concentration (mg/L)	Time of exposure	Mean of mortality	SE	95% Confidence interval	
					Lower bound	Upper bound
*Culex vishnui* group	25	24	62.68	0.92	55.16	70.16
		48	68.00	0.92	60.52	75.48
		72	84.00	0.92	76.52	91.48
	45	24	76.00	0.92	68.52	83.48
		48	80.00	0.92	72.52	87.48
		72	85.32	0.92	77.84	92.84
	60	24	80.00	0.92	72.52	87.48
		48	92.00	0.92	84.52	99.48
		72	97.32	0.92	89.84	104.8
*Anopheles subpictus*	25	24	68.00	0.92	60.52	75.48
		48	85.32	0.92	77.84	92.84
		72	90.68	0.92	83.16	98.16
	45	24	81.32	0.92	73.84	88.84
		48	88.00	0.92	80.52	95.48
		72	97.32	0.92	89.84	104.84
	60	24	86.68	0.92	79.16	94.16
		48	94.68	0.92	87.16	102.16
		72	100.00	0.92	92.52	107.48

**Fig. 1:**
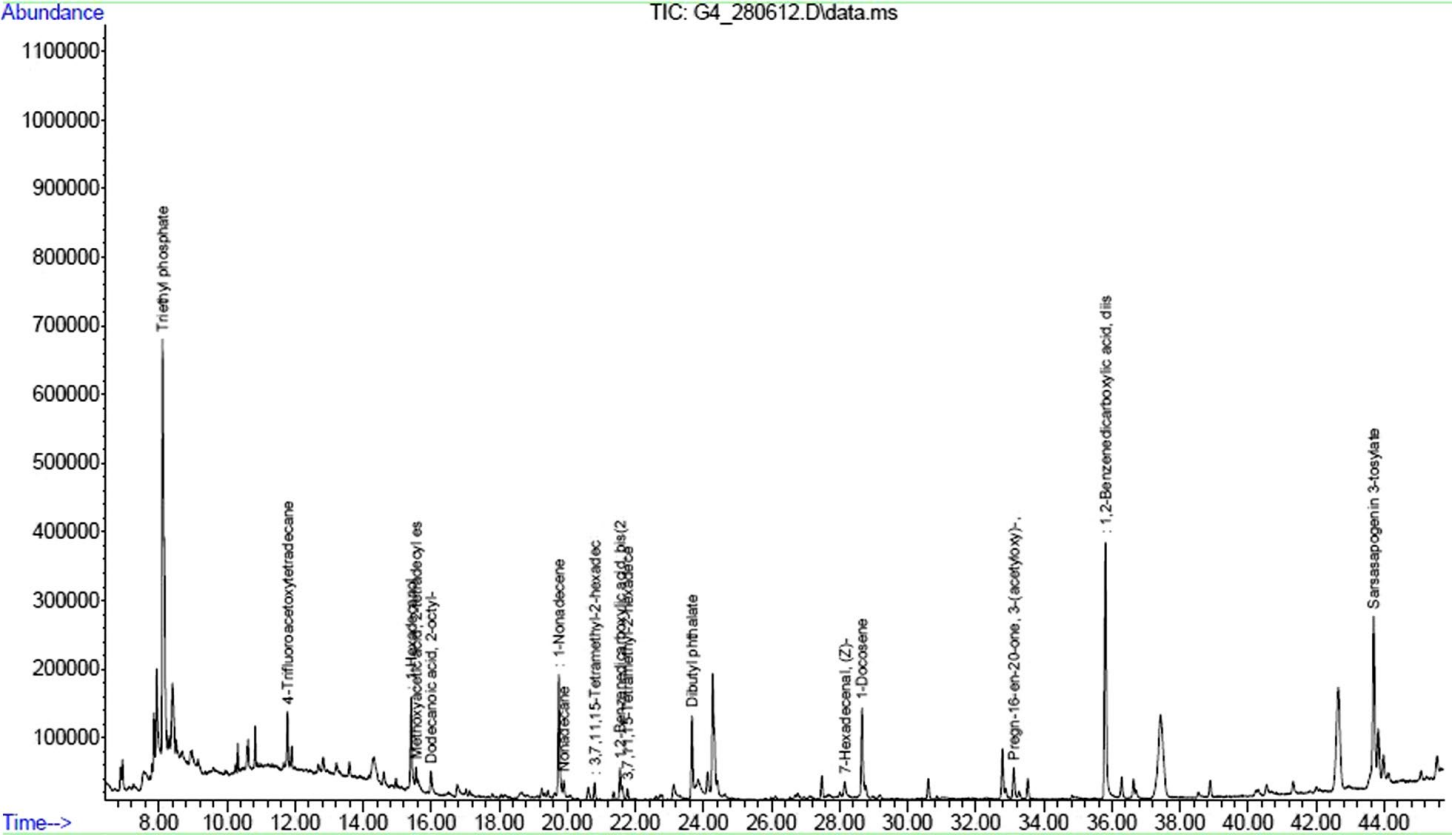
Gas chromatography–mass spectroscopy analysis of bioactive compounds isolated from chloroform: methanol (1:1 v/v) extract of mature leaves of *S. nigrum*

From IR spectroscopy we observed the O–H stretching, a C=C stretching and C=O stretching vibrations of the ester group. The results of the GC–MS analysis showed that at least 18 compounds (Table 5) were present in chloroform: methanol (1:1 v/v) extract of mature leaves of *S. nigrum*. These compounds were identified through mass spectrometry attached with GC (Fig. 1). From the GC–MS analysis seven major bioactive compounds have been identified. The identified compounds were dodecanoic acid (Lauric acid) (peak no. 5, retention time 15.99, mol wt. 200.31), 3,7,11,15-tetramethyl-2-hexadecen-1-ol (phytol) (peak no. 8, retention time 20.80, mol wt. 296.53), 1,2-Benzenedicarboxylic acid or Phthalic acid (peak no. 9, retention time 21.54, mol wt. 166.14), Dibutyl phthalate (peak no. 11, retention time 23.66, mol wt. 278), 1-Hexadecanol (Cetyl alcohol) (peak no. 13, retention time 28.14, mol wt. 242.44), Pregn-16-en-20-one (peak no. 16, retention time 33.11, mol wt. 316.47), Sarsasapogenin 3-tosylate (peak no. 18, retention time 43.68, mol wt. 570.82). From the above prediction phytosteroid compounds from mature leaves of *S. nigrum* may be considered as a potent source as a new natural mosquito larvicidal agent, which was effective against both *Cx. vishnui* group and *An. subpictus.* The LC_50_ concentration of the bioactive compound for third instar larvae was shown to have slightly toxic to the *Daphnia* sp. (2% after 72 h) (Table 6). But it was noted to be non-toxic to the *Diplonychus annulatum* even after 72 h of exposure.

**Table 5:** List of 18 compounds which were present in chloroform: methanol (1:1 v/v) extract of mature leaves of *Solanum nigrum*

Peak	Retention time	Area	Area (%)	Name of the compounds
1	8.107	2,301,900	29.215	Triethyl phosphate
2	11.786	201,861	2.562	4-Trifluoroacetoxytetradecane
3	15.408	315,269	4.001	1-Hexadecanol
4	15.558	55,573	0.705	Methoxyacetic acid
5	15.991	150,057	6.52	Dodecanoic acid
6	19.746	423,197	5.371	1-Nonadecene
7	19.896	44,224	0.561	Nonadecane
8	20.804	68,193	0.865	3,7,11,15-Tetramethyl-2-hexad
9	21.544	94,848	1.204	1,2-Benzenedicarboxylic acid
10	21.771	33,263	0.422	3,7,11,15-Tetramethyl-2 hexadec
11	23.664	357,961	4.543	Dibutyl phthalate
12	24.268	739,637	9.387	1-Nonadecene
13	28.143	79,026	1.003	7-Hexadecenal
14	28.652	432,164	5.485	1-Docosene
15	32.780	214,734	2.725	1-Docosene
16	33.110	154,885	1.966	Pregn-16-en-20-one
17	35.803	154,885	16.801	1,2-Benzenedicarboxylic acid
18	43.686	888,498	11.277	Sarsasapogenin 3-tosylate

**Table 6:** Effect of bioactive compound on few non-target organisms at laboratory condition

Non target organisms	Exposures (hrs)		
	24 (M% ± SE)	48 (M% ± SE)	72 (M% ± SE)
*Daphnia* sp.	0.00 ± 0.00	0.00 ± 0.00	2.00 ± 0.58
Control	0.00 ± 0.00	0.00 ± 0.00	0.00 ± 0.00
*Diplonychus annulatum*	0.00 ± 0.00	0.00 ± 0.00	0.00 ± 0.00
Control	0.00 ± 0.00	0.00 ± 0.00	0.00 ± 0.00

*M* mortality, *SE* standard error

## Discussion

The plant world comprises a rich untapped pool of phytochemicals that may be widely used in place of synthetic insecticides in the mosquito control programme. The search is underway to find out newer insecticides which will be effective, safe and also easily available at lower cost. A wide selection of trees and shrubs has been found to contain phytochemicals that may be of use in the control of mosquitoes. Plants and plant parts have been provided as a good source of novel drug compounds, as plant derived drugs have made the largest contribution to human health. The use of plant extracts, as well as other alternative forms of medical treatment, is enjoying great popularity in the late 1990s. Kishore et al. [18] reviewed the efficacy of phytochemicals against mosquito larvae according to their chemical nature. The physical and spectral data of the present compound are in agreement with those of the values reported in the literature [28, 29]. In the present study, the final active ingredients identified seem to be phytosteroid compounds which are effective against early 3rd instar larvae of the mosquitoes i.e., *Cx. vishnui* group and *An. subpictus* having LC_50_ values 5.64 and 3.68 mg/L respectively after 72 h of exposure. The active ingredients established here are cost effective plant products as *S. nigrum* is abundant and easily available more or less throughout the world. The product is also safe for aquatic ecosystem as it do not cause any ill effect on the health of non target organisms.

Many researchers successfully stated that phytochemicals play a major role to decrease mosquito population through larviciding [30, 31]. Previously several studies reported the effect of phytosteroid on mosquito larvae. Ghosh et al. [32] also reported a steroidal compound from the leaves of *Cestrum diurnum* against *An. subpictus*. The LC_50_ value of the active ingredient was determined as 0.70, 0.89, 0.90 and 1.03 mg/100 mL, for 1st, 2nd, 3rd and 4th instars larvae respectively in the 24 h study period. Chowdhury et al. [33] reported the efficacy of Chloroform: methanol extract (1:1v/v) of mature leaves of *Solanum villosum*, where LC_5O_ values for all instars were between 24.20 and 33.73 ppm after 24 h and between 23.47 and 30.63 ppm after 48 h of exposure period. Rahuman et al. [34]. reported β-sitosterol from *Abutilon indicum*, one kind of phytosteroid as a new active ingredient having larvicidal activity which was effective against *Aedes aegypti*, *An. subpictus* and *Cx. quinquefasciatus* where LC_50_ values were 11.49, 3.58 and 26.67 ppm, respectively after 24 h of exposure. Banerjee et al. [35]. studied the effect of chloroform: methanol (1:1v/v) extracts of *Limonia acidissima* mature leaves against the larval form of *Cx. quinquefasciatus*. The mature leaves of the plant contained steroids as bioactive compounds which showed excellent result having LC_50_ values 1.73, 5.01, 17.37 and 29.19 ppm against all the instars of *Cx. quinquefasciatus* larvae respectively after 72 h of exposure.

However, the present study evaluated the selective toxicity of chloroform: methanol (1:1 v/v) extract of mature leaves of *S. nigrum* against the early 3rd instar larvae of *Cx. vishnui* group and *An. subpictus.* From GC–MS analysis six major compounds have been identified and of them; 1, 2-Benzenedicarboxylic acid [36], dibutyl phthalate [37], phytol, Lauric acid, 3,7,11,15-Tetramethyl-2 hexadec, 7-Hexadecenal [38] have been previously reported for their mosquito larvicidal activity. Thus, during the present study, the identified steroid compounds or the synergistic or potentiating activity may be responsible for larval mortality in the bioassay experiment.

## Conclusion

In conclusion, *S. nigrum* offers a potential larvicidal activity against *Cx. vishnui* group and *An. subpictus*. Further studies are required to know the mode of action of active ingredients. The above findings suggest that this bioactive compound can be used as prototypes for larvicidal agents in the mosquito larval habitats.

## Abbreviations

V/V: volume by volume
GCMS: gas chromatography mass spectrometry
IR: infrared
TLC: Thin layer chromatography
Rf: distance of the spot centre from the start point/distance of the solvent run from the start point
LC_50_: lethal concentration at which 50% of target organism is died
LC_90_: lethal concentration at which 90% of target organism is died
JE: Japanese encephalitis
KOH: potassium hydroxide
FTIR: Fourier transmission infrared spectroscopy
eV: electron volt
NTO: non target organism
n = 9: replication of experiment for nine times
n = 3: replication of experiment for three times
ANOVA: analysis of variance
mol wt.: molecular weight
no.: number
ppm: parts per million
h: hours
SE: standard error
LCL: lower confidence limit
UCL: upper confidence limit
R: regression coefficient value
df: degree of freedom
M%: percentage mortality
F value: it is the ratio of two mean square values
P value: in statistics, the P value is a function of observed sample results (a statistic) that is used for testing a statistical hypothesis
NS: not significant
